# FDA Proposes to Ban Cephalosporins from Livestock Feed

**DOI:** 10.1289/ehp.120-a106

**Published:** 2012-03-01

**Authors:** Charles W. Schmidt

**Affiliations:** Charles W. Schmidt, MS, an award-winning science writer from Portland, ME, has written for *Discover Magazine*, *Science*, and *Nature Medicine*.

The recent ban by the U.S. Food and Drug Administration (FDA) on certain uses of cephalosporin antibiotics in food animals^1^ has not won the agency much favor. Industry groups blasted the rule for intruding on veterinary practice, while food safety advocates said it was at best a minor step toward addressing broader problems with antibiotic resistance.

In issuing the rule on 6 January 2012, the FDA cited declines in the prevalence of cephalosporin-resistant *Salmonella* Heidelberg isolates in chicken meat and in humans following voluntary restrictions on the drugs in Canada.^2^ The Canadian restrictions were limited to use in poultry.^3^ However, according to Chuck Hofacre, a veterinarian and professor at the Center of Food Safety at the University of Georgia, U.S. poultry producers have used cephalosporins very little since 2008, which is when the FDA first tried to restrict the drugs’ use in food animals.^4^ Today, cattle account for most veterinary prescriptions for antibiotics in this country, generally for treating pneumonia, foot rot, and mastitis, according to Gatz Riddell, a veterinarian and executive vice president of the American Association of Bovine Practitioners.

The new rule applies to ceftiofur, 1 of 2 varieties of cephalosporins approved by the FDA for animal use.^1^ Ceftiofur is a β-lactam antimicrobial that targets many bacterial pathogens. Some of these bacterial targets adapt by producing enzymes called β-lactamases, which inactivate cephalosporins and allow the pathogens to survive drug treatment. As of 2009, the prevalence of ceftiofur-resistant *Salmonella* isolates ranged from 4.2% in swine to 14.5% in cattle.^5^

Resistant pathogens may transfer β-lactamases to otherwise drug-sensitive microbes. According to Scott McEwen, a veterinarian at the University of Guelph, Ontario, animal pathogens that contain β-lactamases can also transfer through food to humans. Use of ceftiofur might therefore accelerate resistance to ceftriaxone, a first-line cephalosporin for treating human salmonellosis.

In supporting the FDA’s rule, Caroline Smith De Waal, director of food safety at the consumer advocacy group Center for Science in the Public Interest, cites a study from the Netherlands showing that β-lactamase genes in chicken meat and human tissue samples were nearly identical.^6^ “This shows that resistant pathogens are crossing over from the food supply to human populations,” she says.

**Figure:**
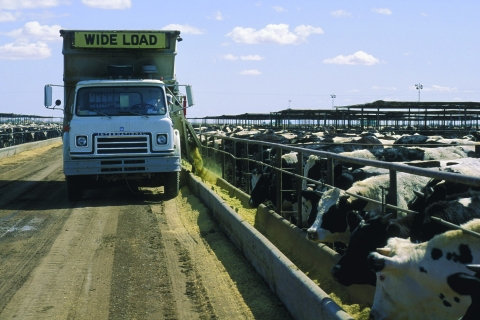
A new FDA ban on cephalosporin use in food animals would largely apply to cattle. © Matt Meadows

The FDA’s new rule revises a rule issued in 2008, which was quickly withdrawn under public pressure—some commenters felt the rule was too broad, others that it failed to meet legal requirements for a ban.^1^ The older rule prohibited all “extralabel” uses of the drugs—that is, dosing regimens or use against diseases for which the treatment has not been explicitly approved. Under the new rule, however, ceftiofur can be used on “minor” species (e.g., sheep and goats) that don’t appear as approved species on the label, and they can also be used for treating extralabel diseases among “major” species (cattle, swine, chickens, and turkeys) as long as the treatment follows the labeled dose, duration, and route of administration for that species and production class of animal.

McEwen says detailed data are not readily available for veterinary drug sales and use in North America. Therefore, the proportion of cephalosporin prescriptions affected by the new rule can’t be easily discerned. And that complicates efforts to predict the impact on cephalosporin resistance trends. “We need to do a better job monitoring the volumes of drugs going towards particular uses, or otherwise we’re just guessing,” McEwen says. “It’s hard to know which applications to target from a resistance standpoint.”

Still, H. Morgan Scott, a veterinarian and professor at the College of Veterinary Medicine at Kansas State University, cautions against focusing entirely on food animals as the principal cause of the antibiotic resistance problem. “There are selection pressures and other ecological events occurring within and among human hosts that are also important in driving the exchange of resistance genes among gut bacteria,” he says. Hofacre agrees, saying, “Usage in humans by medical doctors may be the greatest selection pressure for maintaining these resistance genes in humans.”
